# Do sexual health campaigns work? An outcome evaluation of a media campaign to increase chlamydia testing among young people aged 15–24 in England

**DOI:** 10.1186/1471-2458-13-484

**Published:** 2013-05-17

**Authors:** Maya Gobin, Neville Verlander, Carla Maurici, Angie Bone, Anthony Nardone

**Affiliations:** 1 Field Epidemiology Service, Public Health England, Bristol, UK; 2 Statistics, Modelling and Economics Department, Public Health England Colindale, London, UK; 3 National Chlamydia Screening Programme, Public Health England Colindale, London, UK; 4 HIV & STI Department, Public Health England Colindale, London, UK

**Keywords:** Chlamydia screening, Mass media campaign, Multi media campaigns

## Abstract

**Background:**

A national multimedia campaign was launched in January 2010, to increase the proportion of young people tested for chlamydia. This study aimed to evaluate the impact of the campaign on the coverage and positivity within the National Chlamydia Screening Programme (NSCP) in England.

**Method:**

An interrupted time series of anonymised NCSP testing reports for England for a 27 month period (1st April 2008 to 30th June 2010) was analysed. Reports were assigned to a pre-campaign, campaign and post campaign phase according to the test date. Exclusion criteria included tests for clinical reasons, contacts of known cases, and tests returned from prisons or military services.

Negative binomial and logistic regression modelling was used to provide an estimate for the change in coverage and positivity, during, and after the campaign and estimates were adjusted for secular and cyclical trends.

**Results:**

Adjusting for cyclical and secular trends, there was no change in the overall testing coverage either during (RR: 0.91; 95% CI: 0.72-1.14) or after (RR: 0.88; 95%CI: 0.69-1.11) the campaign. The coverage varied amongst different socio-demographic groups, testing of men increased during the campaign phase while testing of people of black and other ethnic groups fell in this phase. The positivity rate was increased during the campaign (OR: 1.18; 95% CI 1.13-1.23) and further increased in the post-campaign phase (OR: 1.40; 95% CI 1.30-1.51). The proportion of chlamydia infections detected increased for all socio-demographic and self-reported sexual behaviour groups both during and after the campaign.

**Conclusion:**

The uptake of chlamydia testing rose during the campaign; however, this apparent increase was not maintained once overall trends in testing were taken into account. Nonetheless, once secular and cyclical trends were controlled for, the campaign was associated with an increased positivity linked to increased testing of high risk individuals groups in the target population who were previously less likely to come forward for testing. However, our study indicated that there may have been a disparity in the impact of the campaign on different population groups. The content and delivery of ongoing and future information campaigns aimed at increasing chlamydia screening should be carefully developed so that they are relevant to all sections of the target population.

## Background

Genital *Chlamydia* trachomatis is the most commonly diagnosed bacterial sexually transmitted infection (STI) in the UK. The prevalence is highest in sexually active adults aged under 25 years [1,2]. Infections are primarily asymptomatic but if left untreated can result in serious long term complications [3].

In 2003, the National Chlamydia Screening Programme (NCSP) was established in England to increase the early detection and treatment of asymptomatic infection in sexually active people aged under 25 years, in a range of settings [4]. Since its inception, there has been an increase in the proportion of young people being screened for chlamydia, and in 2010 it was estimated that 25% of all 15–24 year olds were tested for chlamydia [5]. However, the proportion of people screened has remained below the estimated 35% coverage which, at the time of the study, was deemed necessary to significantly reduce the prevalence of chlamydia [6,7].

Mass media campaigns have been used for a range of health-related areas and have successfully achieved desirable changes in behaviour and the use of healthcare services amongst young people [8-11]. In January 2010, the mass media campaign, “Chlamydia. Worth talking about”, was launched nationally with the aim of increasing the proportion of people aged 15–24 years having a chlamydia test in NCSP. The campaign sought to normalise conversations about the transmission of chlamydia, raise awareness of the risk of untreated infection and explain the process of diagnosis and treatment. The mass-media campaign with national TV, radio, on-line and poster advertising ran for a total of four weeks and resources (leaflets, posters and access to logos) were made available for local campaigns which continued throughout February and March [12]. The campaign material consisted of short faceless dialogues about chlamydia infection, diagnosis and treatment set in everyday situations and voiced by young people from a range of socio-demographic groups. The uptake of screening amongst 15–24 year olds was identified by the campaign developers as an indicator to evaluate the campaign.

To date, a qualitative evaluation of the campaign, based on pre and post campaign interviews with 1400 children and young people found an increase in the awareness of and testing for chlamydia, however, it did not assess actual changes in chlamydia screening uptake [13]. The aim of this study was to evaluate quantitatively, using an interrupted time series analysis, the immediate impact of the campaign on the coverage and test positivity within the NSCP by socio-demographic characteristics and self-reported sexual behaviour.

## Methods

We undertook retrospective analysis using anonymised data routinely collected from the NCSP over a 27 month period (1st April 2008 to 30th June 2010). Each test record included information on the clinical source of the sample, test result, patient’s clinical history, area of residence, socio-demographic information (ethnicity, age and sex) and self-reported sexual behaviour.

A unique patient identifier based on post code of residence, date of birth and sex was created to de-duplicate the dataset. Socio-economic status for each patient was based on the overall indices of multiple deprivation (IMD) 2007 rank of their postcode of residence [14]. Patients were assigned to one of 10 Strategic Health Authority (SHA) areas according to the Primary Care Trust (PCT) code. Within the NCSP dataset, patients are assigned to one of 22 ethnic groups, which we redefined into four groups: “White”, “Black”, “Asian” and “Other”. Patient age was categorised as 15–19 and 20–24 years. Patients in the NCSP data were assigned to one of three sexual behaviour risk groups according to their response to two questions on sexual behaviour associated with higher risks of chlamydia infection; new sexual partner in last three months and two or more sexual partners in last 12 months. Patients were defined as “High risk” if at least one question was yes, “Low risk” if both questions were no and “Unknown risk” if both questions were unanswered or if either question is unanswered when the other was recorded as no [15].

The patients were assigned to a pre-campaign phase (1st April 2008 to 31st December 2009), campaign phase (1st January to 31st March 2010) and post campaign phase (1st April to 30th June 2010) according to the date of their tests. Patients were excluded from all analyses if tests were for clinical reasons, contacts of known cases, and from prisons or military services.

The NCSP is hosted by the Public Health England and permission for the use of the NSCP data was sought through the NCSP Programme. Ethical approval was not needed.

### Coverage

Coverage was calculated as the number of tests performed in each month using as the denominators mid 2007 population estimates of ethnicity by sex and age-group for each SHA publically available from the Office for National Statistics (ONS) [16]. The population estimate of ethnicity by socio-economic class was not available and we assumed that the distribution of socio-economic class by sex and for each age-group for the whole SHA population was the same for each ethnic group. Records with missing sex, ethnic group, socio-economic class, SHA or age group were excluded from the analysis because of the unavailability of a comparable population denominator. The SHA denominator population is assumed to be constant throughout the study period.

### Positivity

Positivity was defined as the proportion of positive tests in each month. Patients with missing, unknown or equivocal test results were excluded from the analysis.

### Statistical analysis

An interrupted time series analysis was performed in three stages. Stage I: crude estimates were obtained by ignoring all other possible sources of variation in the data. Stage II: secular and cyclical (seasonal) trends were then incorporated to estimate the effect of the campaign after allowing for time trends and seasonality. Stage III analysis extended this to a multivariable mixed effects model by adding SHA as a random effect to allow for the possibility of significant variation between SHAs and the SHA differences were not of interest. The remaining variables as fixed effects and interaction of the socio-demographic fixed effects with phase.

In both stage II and III analyses, month (each taking one of twenty-seven different values) and quarter (each taking one of four different values) were taken as continuous and categorical variables, respectively. The appropriate form of the secular trend was ascertained by assuming a cubic trend and reducing complexity in a stepwise manner when simpler trends did not fit significantly worse (p ≥ 0.05). This was followed, in stage III, by removing non-significant interactions one at a time. The final model was reached when all main effects and interactions were significant.

Coverage, analysis involved negative binomial regression with number of tests as the outcome and the population as the offset (denominator). For positivity, logistic regression was used with number of positive tests as outcome and total number of unequivocal tests as the denominator. Likelihood ratio testing was used to obtain p-values, except where indicated when Wald testing was employed. Stage III estimates and their 95% confidence intervals for variables not involved in interactions were obtained from the final model. The remainder were obtained from single interaction models. Estimates were risk ratios (RR) for coverage and odds ratios (OR) for positivity.

A sensitivity stage I and II analysis of including into the coverage and excluding from the positivity those individuals with unknown socio-demographics was performed as a check on robustness of results.

Given that people could have re-attended for screening during the 27 months under investigation, lag analyses using one and two month time lags were undertaken to assess the impact of non-independence in our data. The analysis was undertaken in Stata 10.

## Results

### Data

**Figure 1:**
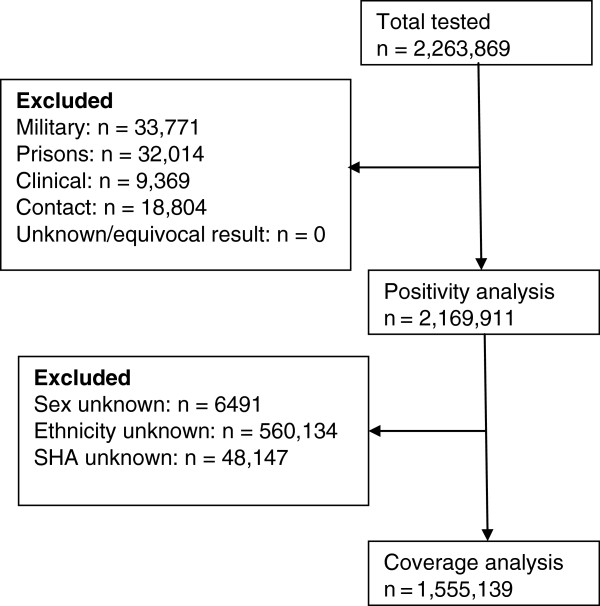
Flowchart of records included in the final coverage and positivity analysis.

Following de-duplication, data on 2,263,869 tests were reported for the 27 months under investigation. After excluding 4% of the records for people tested in military clinics (33,771), prisons (32,014), tests undertaken for clinical reasons (9,369) and contacts of cases (18,804), 2,169,911 records were available for analysis. None of the remaining records had an unknown or equivocal test result and so 2,169,911 records were included in the positivity analysis. A final total of 1,555,139 records were included in the coverage analysis following the exclusion of 28% of the records where the sex, ethnicity or SHA was unknown or missing (Figure 1).

### Coverage

**Figure 2:**
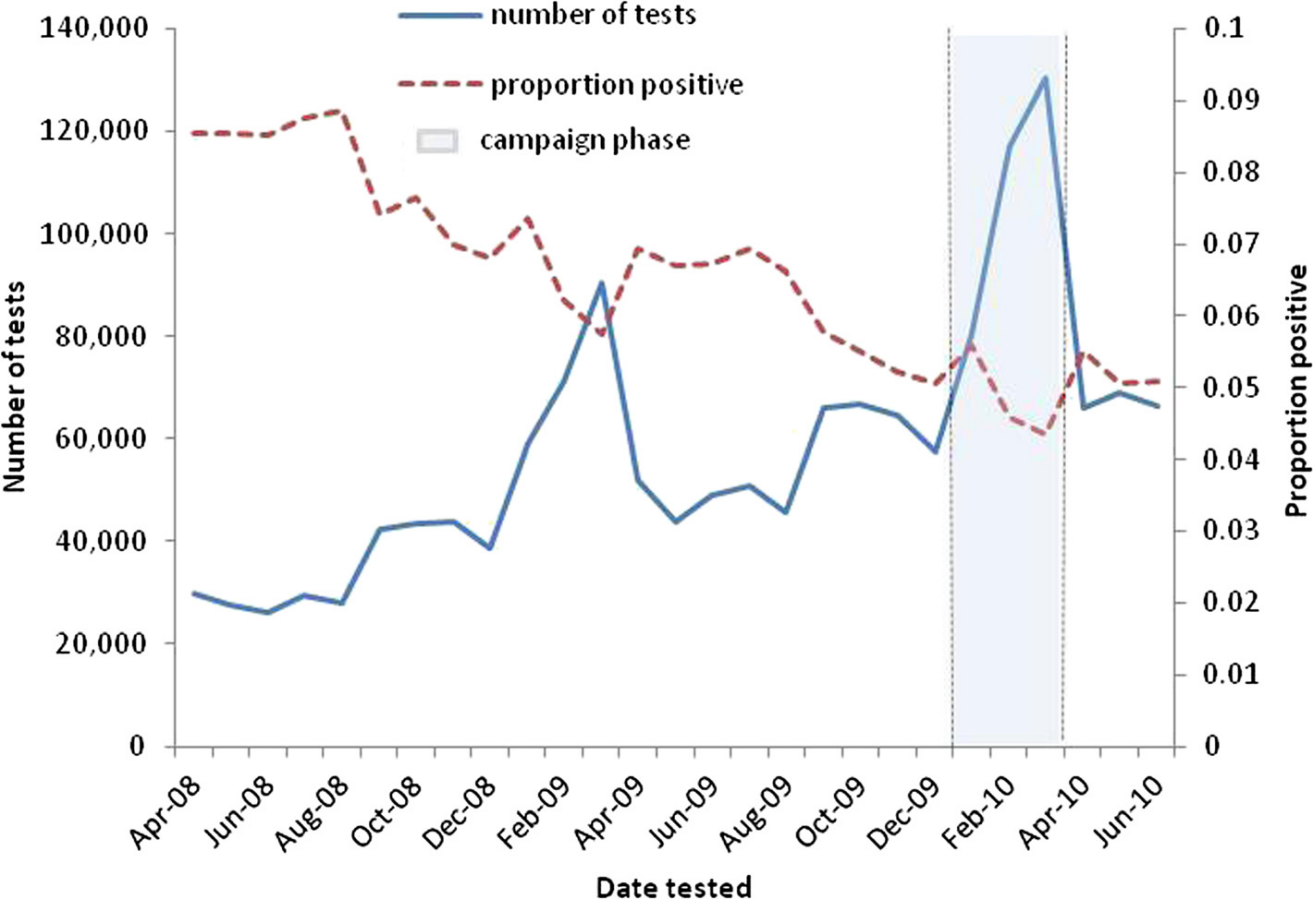
Number of Chlamydia tests and proportion positive from 1st April 2008 to 30th June 2010.

There were secular and cyclical trends in the chlamydia testing observed in the 27 months data collected by the NCSP (Figure 2). The number of people tested for chlamydia increased every month from April 2008 through to July 2010 and there was a considerable increase in quarter 4 (01st January to 31st March) compared to quarter 3 (01st October to 31st December) for both 2008/09 and 2009/10.

The majority of people tested before, during and after the campaign period were female, aged between 15–19 years, of White ethnicity and were from the most deprived social group (Table 1). However, the proportion of males increased during the campaign (from 30.6% pre-campaign to 39.3% during the campaign), while the proportion of people of Black ethnicity fell from 6.2% in the pre-campaign phase to 5.0% during the campaign phase.

**Table 1:** Demographic characteristics of individuals included in each phase for the coverage analyses

**Characteristic**		**Pre – no. tested**	**During – no. tested**	**Post – no. tested**
		**(% of total tested)**	**(% of total tested)**	**(% of total tested)**
**Total Tested**		1025863	327675	201601
		(100)	(100)	(100)
**Sex**	**Male**	313692	128818	70278
		(30.58)	(39.31)	(34.86)
	**Female**	712171	198857	131323
		(69.42)	(60.69)	(65.14)
**Age-group**	**15-19 years**	573332	185464	110161
		(55.89)	(56.60)	(54.64)
	**20-24 years**	452531	142211	91440
		(44.11)	(43.40)	(45.36)
**Socio-economic class**	**1 (most deprived)**	298433	93523	62265
		(29.09)	(28.54)	(30.89)
	**2**	236452	76224	46812
		(23.05)	(23.26)	(23.22)
	**3**	186571	61181	36530
		(18.19)	(18.67)	(18.12)
	**4**	161345	51181	30145
		(15.73)	(15.62)	(14.95)
	**5 (least deprived)**	143062	45566	25849
		(13.95)	(13.91)	(12.82)
**Ethnicity**	**White**	876975	283011	172849
		(85.49)	(86.37)	(85.74)
	**Black**	63885	16247	11519
		(6.23)	(4.96)	(5.71)
	**Asian**	36938	14453	8522
		(3.60)	(4.41)	(4.23)
	**Other**	48065	13964	8711
		(4.69)	(4.26)	(4.32)

*614,772 (28%) Observations excluded because of missing/unknown sex, deprivation, age and ethnicity.

During the campaign there was a crude increase in chlamydia testing compared with the pre-campaign phase (RR: 2.23; 95% CI: 2.22-2.24) which then fell during the post campaign phase, although it remained higher than the pre-campaign period (RR: 1.38; 95% CI: 1.37-1.38) (Table 2). When cyclical and secular trends were adjusted for, there was no change in the overall coverage in both the campaign phase (RR: 0.91; 95% CI: 0.72-1.14) and post campaign phase (RR: 0.88; 95% CI: 0.69-1.11) compared to the pre campaign phase (Table 2).

**Table 2:** Relative change in testing during and after the campaign and by socio-demographic characteristics

		**Campaign Phase**	**RR**	**(95% CI)**	**P value**
**STAGE I**					
Crude RR		Pre	1		<0.001^~^
		During	2.23	(2.22 to 2.41)	
		Post	1.38	(1.37 to 1.38)	
**STAGE II**					
Adjusted RR^x^		Pre	1		0.50^~^
		During	0.91	(0.72 to 1.14)	
		Post	0.88	(0.69 to 1.11)	
**STAGE III**					
**Sex**^**x**^	**Male**	Pre	1		<0.001^~^
		During	1.10	(1.02 to 1.19)	
		Post	0.91	(0.84 to 0.99)	
	**Female**	Pre	1		
		During	0.71	(0.66 to 0.77)	
		Post	0.72	(0.66 to 0.79)	
**Age-group**^**x**^	**15-19 years**		1		<0.001*
	**20-24 years**		0.67	(0.65 to 0.69)	
**Socio-economic class**^**x**^	**1 (most deprived)**		1		<0.001*
	**2**		0.59	(0.57 to 0.62)	
	**3**		0.38	(0.36 to 0.39)	
	**4**		0.27	(0.26 to 0.28)	
	**5 (least deprived)**		0.23	(0.22 to 0.24)	
**Ethnicity**^**x**^	**White**	Pre	1		<0.001^~^
		During	0.91	(0.83 to 1.00)	
		Post	0.79	(0.72 to 0.88)	
	**Black**	Pre	1		
		During	0.78	(0.71 to 0.86)	
		Post	0.74	(0.67 to 0.82)	
	**Asian**	Pre	1		
		During	1.12	(1.01 to 1.24)	
		Post	0.98	(0.88 to 1.09)	
	**Other**	Pre	1		
		During	0.81	(0.74 to 0.90)	
		Post	0.75	(0.67 to 0.83)	

Legend: *RR*, Risk ratio; *CI*, Confidence interval ^x^ adjusted for cyclical and secular trends, *Wald test for socio-demographic variable, ~ likelihood ratio test for interaction in stage III, phase in stages I and II.

Coverage for males and females and for the four ethnic groups varied between the three phases, but did not vary across the phases by age group or socio-economic status. Compared to the pre-campaign phase, males were more likely to be screened during the campaign (RR: 1.09; 95% CI: 1.02-1.19) but less likely in the post campaign phase (RR: 0.91; 95% CI: 0.83-0.99).

The coverage by ethnicity also varied during and after the campaign compared to the pre-campaign phase. Individuals of Black (RR: 0.78; 95% CI: 0.71-0.86) and Other (RR: 0.81; 95% CI: 0.74-0.90) ethnic groups were less likely to be tested during the campaign, and did not differ in the post campaign phase. People of Asian origin were more likely to be tested during the campaign (RR: 1.116; 95% CI: 1.008-1.235) but less likely, although not significantly, in the post campaign period (RR 0.98; 95% CI: 0.88-1.09). Those of White ethnicity were less likely to be screened in the post campaign phase as compared to the pre-campaign phase (RR: 0.79; 95% CI: 0.72-0.88).

### Positivity

Positivity also demonstrated secular and cyclical trends; there was an overall decline in the positivity during the 27 months with a greater drop in quarter 4 as compared with quarter 3 for both 2008/09 and 2009/10 (figure 2). A higher percentage of females, people aged 20–24 years, of Black ethnicity, from the most deprived social group and high risk sexual behaviour group tested positive for chlamydia before, during and after the campaign. The percentage of people testing positive for all sub groups decreased during the campaign phase and remained lower in the post campaign phase as compared with the pre-campaign phase (Table 3).

**Table 3:** Demographic characteristics of individuals included in each phase for the positivity analyses

**Characteristic**		**Pre – no. tested**	**During – no. tested**	**Post – no. tested**
		**(% positive)**	**(% positive)**	**(% positive)**
**Total tested**		1417873	468009	284029
		(6.65)	(4.72)	(5.22)
**Sex**	**Male**	458812	193775	104740
		(5.60)	(3.68)	(4.32)
	**Female**	954824	272873	178396
		(7.16)	(5.47)	(5.75)
	**Sex unknown**	4237	1361	893
		(6.09)	(4.63)	(3.70)
**Age-group**	**15-19 year**	808334	272737	158652
		(6.56)	(4.43)	(5.00)
	**20-24 years**	609539	195272	125377
		(6.78)	(5.14)	(5.49)
**Socio-economic class**	**1 (most deprived)**	403063	129909	87207
		(7.89)	(5.59)	(5.90)
	**2**	325252	108604	64290
		(6.82)	(4.82)	(5.36)
	**3**	253380	87668	50397
		(6.09)	(4.42)	(4.92)
	**4**	212359	71750	41427
		(5.85)	(4.21)	(4.94)
	**5 (least deprived)**	186474	63326	36255
		(5.16)	(3.79)	(4.20)
	**unknown**	37345	6752	4453
		(7.56)	(4.80)	(4.11)
**Ethnicity**	**White**	905090	287450	175135
		(6.94)	(5.06)	(5.58)
	**Black**	66128	16785	12042
		(8.25)	(6.61)	(7.51)
	**Asian**	37882	14763	8778
		(2.28)	(1.66)	(1.88)
	**Other**	49532	14260	8934
		(7.64)	(6.00)	(6.63)
	**unknown**	359241	134751	79140
		(5.95)	(3.98)	(4.29)
**Sexual behaviour**	**High risk**	552570	152441	93649
		(8.81)	(6.87)	(7.16)
	**Low risk**	330365	89744	50719
		(4.66)	(3.28)	(4.00)
	**unknown**	534938	225824	139661
		(5.65)	(3.85)	(4.36)

The crude overall positivity rate was lower in the campaign phase as compared with the pre-campaign phase (OR: 0.70; 95%CI: 0.69-0.71) (Table 4). The positivity rate increased in the post campaign phase but remained lower than the pre-campaign period (OR: 0.77; 95% CI: 0.76-0.79). Adjusting for secular and cyclical trends, the positivity rate during the campaign was higher than the pre-campaign phase (OR: 1.18; 95% CI 1.13-1.23) and further increased in the post-campaign phase (OR: 1.40; 95% CI 1.30-1.51) (Table 4).

**Table 4:** Relative change in positivity of Chlamydia testing during and after the campaign and by socio-demographic characteristics

		**Campaign Phase**	**OR**	**(95% CI)**	P value*
**STAGE I**					
**Crude OR**		Pre	1		<0.001
		During	0.70	(0.67 to 0.71)	
		Post	0.77	(0.76 to 0.79)	
**STAGE II**					
**Adjusted OR**^**x**^		Pre	1		<0.001
		During	1.18	(1.13 to 1.23)	
		Post	1.40	(1.30 to 1.51)	
**STAGE III**					
**Sex**^**x**^	**Male**	Pre	1		<0.001
		During	1.04	(0.99 to 1.10)	
		Post	1.30	(1.20 to 1.41)	
	**Female**	Pre	1		
		During	1.19	(1.14 to 1.25)	
		Post	1.35	(1.25 to 1.46)	
	**unknown**	Pre	1		
		During	1.26	(0.88 to 1.81)	
		Post	0.85	(0.50 to 1.43)	
**Age-group**^**x**^	**15-19 years**	Pre	1		<0.001
		During	1.10	(1.05 to 1.15)	
		Post	1.31	(1.21 to 1.42)	
	**20-24 years**	Pre	1		
		During	1.20	(1.15 to 1.26)	
		Post	1.37	(1.27 to 1.48)	
**Socio-economic class**^**x**^	**1 (most deprived)**	Pre	1		0.005
		During	1.13	(1.08 to 1.19)	
		Post	1.30	(1.20 to 1.41)	
	**2**	Pre	1		
		During	1.13	(1.07 to 1.19)	
		Post	1.34	(1.24 to 1.46)	
	**3**	Pre	1		
		During	1.16	(1.10 to 1.22)	
		Post	1.36	(1.25 to 1.48)	
	**4**	Pre	1		
		During	1.16	(1.09 to 1.23)	
		Post	1.44	(1.32 to 1.57)	
	**5 (least deprived)**	Pre	1		
		During	1.17	(1.10 to 1.25)	
		Post	1.36	(1.24 to 1.49)	
	**unknown**	Pre	1		
		During	1.06	(0.93 to 1.20)	
		Post	0.98	(0.81 to 1.17)	
**Ethnicity**^**x**^	**White**	Pre	1		<0.001
		During	1.15	(1.10 to 1.20)	
		Post	1.35	(1.25 to 1.46)	
	**Black**	Pre	1		
		During	1.25	(1.15 to 1.35)	
		Post	1.57	(1.41 to 1.75)	
	**Asian**	Pre	1		
		During	1.25	(1.05 to 1.49)	
		Post	1.40	(1.13 to 1.74)	
	**Other**	Pre	1		
		During	1.25	(1.14 to 1.37)	
		Post	1.46	(1.29 to 1.65)	
	**unknown**	Pre	1		
		During	1.08	(1.03 to 1.14)	
		Post	1.23	(1.13 to 1.33)	
**Sexual behaviour**^**x**^	**High risk**	Pre	1		<0.001
		During	1.21	(1.16 to 1.27)	
		Post	1.36	(1.25 to 1.46)	
	**Low risk**	Pre	1		
		During	1.10	(1.04 to 1.17)	
		Post	1.42	(1.30 to 1.55)	
	**unknown**	Pre	1		
		During	1.08	(1.03 to 1.13)	
		Post	1.28	(1.18 to 1.39)	

Legend *OR*, Odds ratio; *CI*, Confidence interval ^x^ adjusted for cyclical and secular trends ^*^likelihood ratio test for interaction in stage III, phase in stages I and II.

The positivity rate increased in the campaign and post campaign phase for all sub groups analysed. The following sub-groups had a greater increase in proportion testing positive during the campaign as compared with the pre-campaign phase: women (OR: 1.19; 95% CI 1.14 – 1.25), people aged 20–24 years old (OR: 1.20; 95% CI 1.15 – 1.26), in the least deprived social groups (OR: 1.17; 95% CI 1.10 – 1.25), from non-White ethnic groups and people with high risk sexual behaviour (OR: 1.21; 95% CI 1.17-1.27).

## Discussion

Our study found that the overall uptake of testing (coverage) did not appear to be affected by the national campaign, as the apparent increase in testing was not maintained after the secular and cyclical trends in testing were taken into account. However, we found a differential change in the coverage in the various socio-demographic groups both during and after the campaign. Testing in women and people of Black and Other ethnic origins fell during the campaign, while testing of men and people of Asian ethnicity increased. However, the increases in testing in these groups were not sustained after the campaign.

The overall proportion of people testing positive increased during the campaign, an observation that persisted in the immediate post campaign phase after adjusting for secular and cyclical trends in positivity. The campaign was associated with an increase in the positivity of chlamydia infections detected for all socio-demographic and risk groups, including people defined as having high risk sexual behaviour.

Since 2008, there has been a steady increase in the number of people tested for chlamydia through the NCSP associated with previous and ongoing national and local initiatives such as national targets, use of financial incentives and professional education programmes to promote screening [17-20]. In addition, in the last quarter of each financial year (January to March), there have been marked increases in testing observed which have coincided with the requirement for local services to meet national coverage targets set by the Department of Health [20]. However, these initiatives and annual targets have had an equal and opposite effect on positivity, as more low risk individuals were recruited to the NCSP for testing [21]. Our study demonstrates that the increase in the number of people tested during the campaign phase was not greater than what would be expected for that time of year. However, the proportion of people testing positive during the campaign phase was more than expected and resulted in a smaller than anticipated reduction in positivity for January to March 2010.

A review of the positivity within the NCSP during 2007/08 found that women and people of black and mixed ethnicity were more likely to be tested by the programme and attributed this discrepancy in recruitment to the design and delivery of local services and initiatives [20]. In contrast, our results indicate that, unlike previous initiatives, this campaign was associated with an overall higher positivity and may have had some success in targeting groups such as men, people of Asian ethnicity and those at higher risk of having the infection that were previously not typically recruited to the NCSP.

Our study contributes to the limited and inconsistent evidence base of evaluations of mass media campaigns aimed at promoting chlamydia testing. Two studies measuring the impact of multi-media campaigns for chlamydia screening found that both the awareness of and information seeking behaviours of the target populations increased and in one study persisted for weeks after the campaign finished [22,23]. However, neither of the studies ascertained the impact on subsequent testing behaviour. A further three studies have measured the effect of multi-media campaigns on chlamydia testing uptake. Evaluations of campaigns in Denmark and Australia concluded that their campaigns did not increase chlamydia testing [24,25]. While an evaluation of another campaign in Australia found that chlamydia testing increased significantly for both men and women during the media campaign and was closely paralleled by an increase in chlamydia notifications. However, in that study the investigators did not control for cyclical or secular longer-term trends [26].

The timing and type of broadcast, the overall duration of campaigns as well as the integration with other campaigns have been identified as key to the success of public information campaigns and are likely to account for the discrepancy in the impact of the campaigns run to date [27]. The English campaign aimed to promote communication between individuals to encourage safer sexual behaviours and attitudes. Thus, a range of voices were used instead of casting people to deliver the key messages in order to appeal to a wider audience. The qualitative evaluation of the campaign demonstrated an increase in the number of young people aware of chlamydia and who claimed to have had a test as a result of the campaign. However, over two fifths of those interviewed felt that the campaign was not relevant to them, which may explain why we observed differential effects of the campaign on coverage and positivity in the various socio-demographic groups [13].

We have identified a number of limitations with this study. The NCSP dataset analysed includes data from services that are part of the NCSP programme. An estimated two thirds of chlamydia tests are conducted at NCSP services, thus there is the potential that the analysis may have underestimated the effect of the campaign [28].

Our analysis assumed that the changes in testing and positivity that occurred during and after the campaign phase were as a result of campaign only. The use of the already available NSCP dataset meant that we were unable to collect any additional data, such as the motivation for testing from those who were tested. We did not take into account any concurrent local campaigns or incentive schemes aimed at health professionals and/or young people which have previously been used to increase chlamydia screening and which may have been used during the campaign phase to target high risk individuals [29,30].

It is likely that people would have attended for chlamydia testing on more than one occasion in the 27 months from April 2008 to July 2010 and so in addition to de-duplicating our dataset our analysis included an assessment for correlation. This lag analysis produced very similar results to those presented. However, the choice of lag period was limited by the number of months of data available for analysis and it is possible that results could have materially changed if there was correlation for time periods of more than two months and would have been missed by our assessment.

The exclusion of just under 30% of all records from the coverage analysis could potentially bias our results. We believe that this is unlikely as the results from the sensitivity analysis were not substantively different to those presented. A similar conclusion was reached with the sensitivity analysis of the positivity data (Additional file 1). Finally, we also assumed that the distribution of people by socioeconomic group was the same for all ethnic groups. This decision was taken on pragmatic grounds, although there is evidence that some ethnic groups are more likely to live in areas of greater deprivation [31].

## Conclusion

This study adds to the limited evidence base of the effectiveness of media campaigns on chlamydia testing uptake and to date is the only study to assess the effect of the campaign by socio-demographic characteristics and self-reported sexual behaviour. The media campaign, once secular and cyclical trends were controlled for, was associated with an increased testing of high risk individuals and groups in the target population who were previously less likely to come forward for testing.

Our findings corroborate the claims of positive behaviour changes reported in the qualitative evaluation and together indicate that there may have been a disparity in the impact on different population groups. The content and delivery of information campaigns aimed at increasing chlamydia screening therefore need to be carefully developed to ensure that they are relevant to all sections of the target population.

This study also demonstrates the potential value of using routine national surveillance data to determine the effectiveness of population wide initiatives.

## Abbreviations

CI: Confidence interval; IMD: Indices of multiple deprivation; NCSP: National chlamydia screening programme; ONS: Office for national statistics; OR: Odds ratio; PCT: Primary care trust; PR: Public relations; RR: Risk ratio; SHA: Strategic health authority; STI: Sexually transmitted infection; TV: Television; UK: United Kingdom; CYP: Children and young people

## Competing interests

The authors declare that they have no competing interests.

## Authors’ contributions

MG participated in the design of the study, performed the statistical analysis and drafted the manuscript. NV participated in the design of the study, performed the statistical analysis and helped to draft the manuscript. CM helped to draft the manuscript. AB helped to draft the manuscript. AN conceived the study and participated in its design and helped to draft the manuscript. All authors read and approved the final manuscript.

## Pre-publication history

The pre-publication history for this paper can be accessed here:

http://www.biomedcentral.com/1471-2458/13/484/prepub

## Supplementary Material

Additional file 1 Coverage and Positivity of chlamydia testing before during and after the “Chlamydia, Worth Talking About” campaign. Click here for file
